# Optimal population size to detect quantitative trait loci in Korean native chicken: a simulation study

**DOI:** 10.5713/ab.21.0195

**Published:** 2021-08-25

**Authors:** Chiemela Peter Nwogwugwu, Yeongkuk Kim, Sunghyun Cho, Hee-Jong Roh, Jihye Cha, Seung Hwan Lee, Jun Heon Lee

**Affiliations:** 1 Department of Animal Science, University of Calabar, Etagbor, PMB 1115, Calabar, Nigeria; 2 Division of Animal and Dairy Science, Chungnam National University, Daejeon 34134, Korea; 3 Animal Genetic Resources Center, National Institute of Animal Science, RDA, Hamyang 50000, Korea; 4 Animal Genomics and Bioinformatics Division, National Institute of Animal Science, RDA, Wanju 55365, Korea

**Keywords:** Chicken, Heritability, Quantitative Trait Loci (QTL) Detection, Reference Population Size, Simulation

## Abstract

**Objective:**

A genomic region associated with a particular phenotype is called quantitative trait loci (QTL). To detect the optimal F_2_ population size associated with QTLs in native chicken, we performed a simulation study on F_2_ population derived from crosses between two different breeds.

**Methods:**

A total of 15 males and 150 females were randomly selected from the last generation of each F_1_ population which was composed of different breed to create two different F_2_ populations. The progenies produced from these selected individuals were simulated for six more generations. Their marker genotypes were simulated with a density of 50K at three different heritability levels for the traits such as 0.1, 0.3, and 0.5. Our study compared 100, 500, 1,000 reference population (RP) groups to each other with three different heritability levels. And a total of 35 QTLs were used, and their locations were randomly created.

**Results:**

With a RP size of 100, no QTL was detected to satisfy Bonferroni value at three different heritability levels. In a RP size of 500, two QTLs were detected when the heritability was 0.5. With a RP size of 1,000, 0.1 heritability was detected only one QTL, and 0.5 heritability detected five QTLs. To sum up, RP size and heritability play a key role in detecting QTLs in a QTL study. The larger RP size and greater heritability value, the higher the probability of detection of QTLs.

**Conclusion:**

Our study suggests that the use of a large RP and heritability can improve QTL detection in an F_2_ chicken population.

## INTRODUCTION

The application of genomics in agriculture focuses on identifying genes responsible for economically important traits in plants and animals. Some of these traits are characterized by wide variability in the expression of genes at certain loci, i.e., quantitative trait loci (QTL). A genomic region associated with a particular phenotype is called QTL. Classification of the chromosomal regions containing QTLs could be useful in marker-assisted selection to increase breeding efficiency [1]. Also, the combination of a molecular linkage map with powerful statistical approaches enables the genetic partition of complex traits. Chicken has particular advantages in such analysis due to its short life cycle and many offspring [2]. However, several factors could influence detection of QTLs, such as genotyping errors, training population size, phenotypic data replication levels, and various environmental effects. The evaluation of some of them is either difficult or time consuming in practice. As an alternative, simulation experiments are generally performed for the evaluation of such factors [3].

A simulation study allows the testing of several theories, permitting an unravelling of the multifaceted evolutionary patterns that are otherwise difficult to understand. For example, the elucidation of the history of human migration provides significant insight into the present patterns of DNA variation in humans [3–5]. Simulation studies of beef cattle and other livestock have provided information on their potential for genomic evaluation. Studies have included the prediction of total genetic value [6], genomic prediction of simulated multi-breed and purebred cattle [7], genomic selection accuracy in simulated populations [8], and a comparison between single- and two-step genomic best linear unbiased prediction methods in simulated beef cattle [9,10]. The chicken 60K single-nucleotide polymorphism (SNP) panel currently provides a level of genome coverage and map resolution that are unavailable from microsatellite markers. The high density SNP panel also has the potential to achieve improved accuracy in determining QTL locations. An F2 population is useful for detecting QTLs because it is a cross between two populations differing phenotypically in a trait [2]. Ledur et al [11] showed that designed populations, such as F_2_ populations for use in genome-wide association studies (GWAS), had advantages over random populations in terms of reducing the false discovery rate and improving mapping accuracy. Several experiments have been conducted based on this design in different livestock species. The design is especially useful in pigs and chickens because of their shorter generation interval and higher prolificacy than other species. The objective of this study was to investigate the optimal size of an F_2_ population in QTL detection through simulation using QMSim software.

## MATERIALS AND METHODS

### Simulation of F**_2_** population, population structure, and simulation parameters

The number of QTLs was examined in two different F_2_ populations. A total of six chicken populations were simulated, including Line 1 and Line 2, which performed as a typical sire and dam population, respectively. The crossing of males of Line 1 and females of Line 2 produced the F_1a_ population, whereas mating of males of Line 2 and females of Line 1 produced F_1b_ population. Similarly, the males of F_1a_ and females of F_1b_ produced the F_2a_ population, and the females of F_1a_ and males of F_1b_ created the F_2b_ population in this study. However, we did not include the effect of mating system in this study.

The QMSim software package [12] was used for simulation of phenotypic and genotypic datasets of the populations. These simulated datasets mimicked the actual population structures and extent of linkage disequilibrium (LD) existing in the Korean native chicken population [13]. Table 1 summarizes the parameters for simulation. A 50K marker-density panel was simulated to generate bi-allelic markers distributed across 18 autosomal chromosomes of different lengths. In the beginning, a historical population (HP) was simulated, which had a constant size of 10,000 individuals across 1,000 generations. Then, the size was gradually reduced to 8,000 individuals in the subsequent 1,050 generations to create an initial LD and mutation-drift equilibrium. The number of individuals produced for each sex was equal (equal probability of being male or female), and the mating performed among parents was random. For simulating two different pure lines (Lines 1 and 2), 60 males and 600 females were selected from the last generation of the HP. As Line 1 acted as a sire population, individuals selected from this population were based on a higher true breeding value (TBV). Oppositely, Line 2 being the dam population, the selection of individuals from Line 2 was based on a lower TBV. The mating design in each population was based on positive assortative mating. A total of 660 selected individuals was used as the effective population size, Ne was simulated across 20 generations, with each dam producing 10 offspring per generation in all simulations. A total of 330 individuals (30 males and 300 females) were chosen from the last generation of HP and bred for five generations to create two different F_1_ populations (F_1a_ and F_1b_). Finally, 15 males and 150 females were randomly chosen from the last generation of each F_1_ population and randomly bred for six more generations to create two different F_2_ populations (F_2a_ and F_2b_), following a similar mating design as described earlier. The replacement ratio for both sires and dams was 100%. Traits with a phenotypic variance of 1 and heritability levels of 0.1, 0.3, and 0.5 were used in the simulation. Three reference populations (RP) consisting of 100, 500, and 1,000 individuals were created through a random selection of individuals from generations 5 and 6 of F_2_ population.

Our simulated genome comprised 18 pairs of chromosomes, with a length identical to the actual Korean native chicken genome length of 2,729.4 cM [13]. A marker density of 50K was selected to ensure sufficient density for segregating bi-allelic loci. The effect of markers on traits was neutral and the effect of QTL was considered to explain 100% of the genetic variance. The whole-genome consisted of 35 QTLs, where these segregated QTLs consist of 2 to 4 alleles per loci (randomly distributed), with a minor allelic frequency greater than 0.01. The additive genetic effect of the QTL was sampled from a gamma distribution, with a parametric shape equal to 0.4. The rate of missing marker genotype and marker genotyping error was 0.05 and 0.005, respectively. A recurrent mutation rate of 10^−5^ was used for markers and QTLs throughout the simulation to obtain a mutation-drift equilibrium in the population. Phenotypes were generated by adding random residuals to the QTL effects.

### Statistical model for quantitative trait loci detection

The F_2_ population was chosen as the RP as their parents were produced by crossing two different families. In GWAS, all markers are required to be in LD, with causal variants in close proximities. All SNPs were coded as AA = 0, AB = 1, and BB = 2, respectively [14]. The statistical model was as follows:


$$y=\mu +{CG}_{i}+b_{1}{SNP}_{k}+A_{1}+e_{ijk}$$

where y is the phenotype of individuals; μ is the overall mean, *CG_i_* is the vector of fixed contemporary group effect for generation by sex; *b*_1_ is the fixed/random effect of marker genotype; *SNP_k_* is the recoded marker genotype (0, 1, and 2); *A*_1_ is the vector of the random polygenic effect with $\sim \text{N}(0,G\sigma_{a}^{2})$, where G is the additive genomic relationship matrix (GRM) and $\sigma_{a}^{2}$ is the random additive effect of animals, and *e_ijk_* is the random residual effect $\sim \text{N}(0,I\sigma_{a}^{2})$, where I is the identity matrix.

To map QTLs, a modified Bonferroni-type multiple testing correction threshold was used [15] to restrict the experiment-wise error rate to 0.05 [16].

## RESULTS AND DISCUSSION

**Figure 1:**
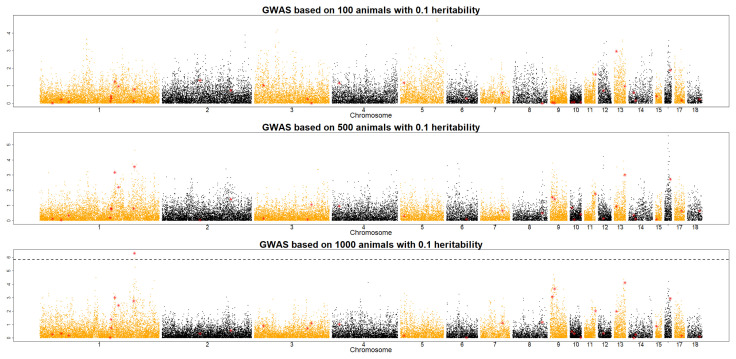
Manhattan plot of QTL detection profiles for an F_2_ chicken population showing the −log10 p-values across the 18 chromosomes with a heritability of 0.1 for RP sizes of 100, 500, and 1,000. Red triangles and dotted lines indicate possible locations of QTLs and the genome-wide significant threshold, respectively. Note that GWAS based on 100 animals with 0.1 heritability should be as (a) RP of 100 with *h*^2^ of 0.1, (b) RP of 100 with *h*^2^ of 0.3, (c) RP of 100 with *h*^2^ of 0.5. QTL, quantitative trait locus; RP, reference population; GWAS, genome-wide association studies.

**Figure 2:**
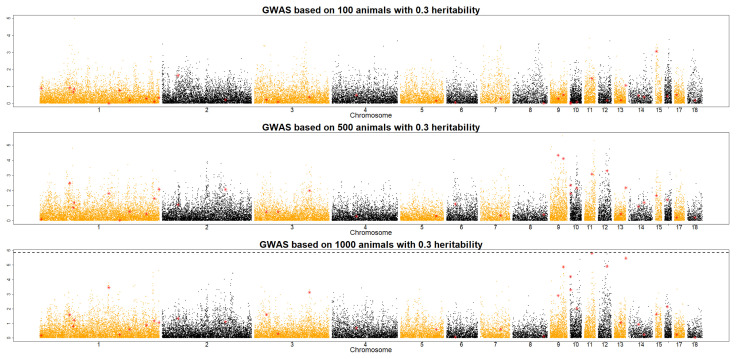
Manhattan plot of QTL detection profiles for an F_2_ chicken population showing the −log10 p-values across the 18 chromosomes with a heritability of 0.3 for RP sizes of 100, 500, and 1,000. Red triangles and dotted lines indicate possible locations of QTLs and the genome-wide significant threshold, respectively. QTL, quantitative trait locus; RP, reference population.

To investigate the optimal size of an F_2_ population in QTL detection, QMSim software was used to simulate data sets derived under different scenarios (e.g., *h*^2^ = 0.1, 0.3, and 0.5; RP size = 100, 500, and 1,000), as shown in Figure 1, 2, and 3. Across the RP sizes, we observed an overall increase in the number of significant QTLs across the different chromosomes.

With a RP size of 100, no QTL was detected to satisfy Bonferroni value at three different heritability levels. In a RP size of 500, two QTLs were detected when the heritability was 0.5. With a RP size of 1,000, 0.1 heritability was detected only one QTL, and 0.5 heritability shows that five QTLs were detected. To sum up, RP size and heritability are playing a key role to detect QTLs in the QTL study. This result implies that RP sizes should be increased in accordance with heritability in an F_2_ chicken population. With a RP size of 1,000, many QTLs were detected at different *h*^2^ levels of traits, even at the *h*^2^ value of 0.1 (Figure 1). The results of this study imply that increasing the RP size and heritability level improved QTL detection in an F_2_ population. However, the optimal RP size for QTL detection should be at least 500 individuals across scenarios of traits with low to high heritability levels (*h*^2^ = 0.1, 0.3, and 0.5) to obtain more significant QTLs in an F_2_ chicken population. These results support an earlier study by Hocking [17], who detected QTLs for production traits in F_2_ crosses between 250 to 700 birds of two breeds. In 1992, the Korean government launched the nationwide Korean native chicken restoration project, which was mainly administered by the National Institute of Animal Science (NIAS) and focused on the development of meat-type native chicken lines [18]. As part of this project, Korean Ogye and White Leghorn cross populations were investigated for the determination of QTLs and eventually, the causative mutations for meat- and egg-related traits. The results of the present study can be used as an initial framework for designing and implementing QTL detection in an F_2_ chicken population, especially cross populations between the Korean Ogye and White Leghorn breeds. However, the population structure and genetic architecture of traits should also be considered to optimize the RP sizes for QTL detection in the chicken industry.

## CONCLUSION

In general, a large RP size (1,000) had a positive effect on QTL detection compared with a RP size of 100 or 500. The RP size and heritability levels should be considered for QTL detection in an F_2_ chicken population.

## Figures and Tables

**Figure 3:**
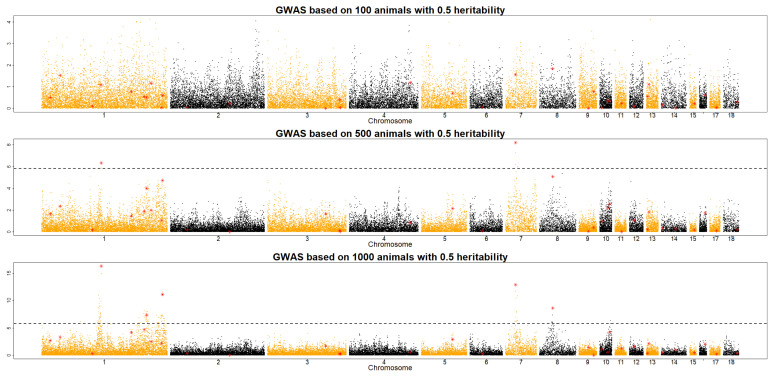
Manhattan plot of QTL detection profiles for an F2 chicken population showing the −log10 p-values along the 18 chromosomes with a heritability of 0.5 for RP sizes of 100, 500, and 1,000. Red triangles and dotted lines indicate possible locations of QTLs and the genome-wide significant threshold, respectively. QTL, quantitative trait locus; RP, reference population.

**Table 1:** Population structure and simulation parameters

Parameter	Value
Step 1: HG	
Number of generations (size) – phase 1	1,000 (10,000)
Number of generations (size) – phase 2	1,050 (8,000)
Number of generations (size) – phase 3	20 (660)
Step 2: Pure-line generations	
Number of founder males from the HG	60
Number of founder females from the HG	600
Number of generations	20
Step 3: Recent generations (F_1_ populations)	
Number of founder males from pure line	30
Number of founder females from pure line	300
Number of generations	5
Step 4: Recent generations (F_2_ populations)	
Number of founder males from F_1_ population	15
Number of founder females from F_1_ population	150
Number of generations	6
Number of offspring per dam	10
Ratio of males	50%
Mating system	Selective
Replacement ratio for males	100%
Replacement ratio for females	100%
Selection	TBV/positive assortative
Ratio of missing sires and dams	5%
Trait heritability	0.1, 0.3, or 0.5
Phenotypic variance	1.0
Genome	
Number of chromosomes	18
Total length	2,729.4 cM
Number of markers	33,802
Marker distribution	Evenly spaced
Number of QTLs	35
QTL distribution	Random
MAF for markers	0.1
MAF for QTL	0.1
Additive allelic effects for markers	Neutral
Additive allelic effects for QTL	Gamma distribution (shape = 0.40)
Rate of missing marker genotypes	0.05
Rate of missing QTL genotypes	0.00
Rate of marker genotyping error	0.005
Rate of recurrent mutation	0.00025
QTL mutation rate	2.5e-005

HG, historical generation; TBV, true breeding value; QTL, quantitative trait locus; MAF, minor allele frequency.
